# Impact of thymidine phosphorylase and CD163 expression on prognosis in stage II colorectal cancer

**DOI:** 10.1007/s12094-022-02839-2

**Published:** 2022-05-14

**Authors:** Donia Kaidi, Louis Szeponik, Ulf Yrlid, Yvonne Wettergren, Elinor Bexe Lindskog

**Affiliations:** 1 Surgical Oncology Laboratory, Department of Surgery, Institute of Clinical Sciences, Sahlgrenska University Hospital/Östra, Sahlgrenska Academy at University of Gothenburg, SU Sahlgrenska, 41345 Gothenburg, Sweden; 2 Department of Microbiology and Immunology, University of Gothenburg, Medicinaregatan 7, 41390 Gothenburg, Sweden; 3 Department of Surgery, Sahlgrenska University Hospital, 416 85 Östra, Sweden

**Keywords:** Macrophages, Thymidine phosphorylase, Colorectal neoplasm, Biomarkers, Microdissection, Microsatellite instability

## Abstract

**Background:**

Tumor-associated macrophages (TAM) are known to facilitate colorectal cancer (CRC) growth. High macrophage infiltration in thymidine phosphorylase (TYMP) expressing CRC may correspond to poor prognosis. The prognostic impact of the expression CD163, a receptor associated with TAM, and TYMP in stroma, respectively, tumor tissue is not yet established. The aim of this study was to identify the potential associations between TYMP and CD163 expression levels and relapse-free survival (RFS) of patients with stage II CRC, and if microdissection is of importance.

**Methods:**

Stage II CRC patients, radically resected with relapse (*n* = 104), were matched to patients with a 5-year relapse-free follow-up (*n* = 206). Gene expression of TYMP and CD163 was analyzed in snap-frozen tumor tissues and in microdissected formalin-fixed tumor tissues separated into tumor epithelium and stroma.

**Results:**

TYMP expression was high in poorly differentiated tumors, right-sided CRC, and tumors with high microsatellite instability CD163-expressing macrophages near tumor epithelial cells had high expression in poorly differentiated and T4 tumors. High TYMP expression in tumor epithelial cells was in the multivariate analyses associated with shorter relapse-free survival (hazard ratio 1.66; 95% confidence interval: 1.09–2.56; *p* < 0.05).

**Conclusions:**

TYMP expression in tumor epithelial cells was associated with RFS and emphasizes the need for tissue microdissection. Additional studies are needed to establish whether *TYMP* and *CD163* could add clinically relevant information to identify high-risk stage II patients that could benefit from adjuvant chemotherapy.

**Supplementary Information:**

The online version contains supplementary material available at 10.1007/s12094-022-02839-2.

## Introduction

Colorectal cancer (CRC) is the third most common cancer worldwide [1]. Its curative treatment is surgery, which sometimes is complemented with chemotherapy based on 5-fluorouracil (5-FU) [2]. Mutations in tumor-suppressor genes, oncogenes, and genes related to DNA repair mechanisms play an important part in the development of CRC, as does the tumor microenvironment with its heterogenous composition of tumor and non-tumor cells [3].

Adjuvant chemotherapy is usually not recommended to patients with stage II CRC unless high-risk features have been identified, including < 12 analyzed lymph nodes, perineural invasion, tumor perforation, poorly differentiated tumors, and T4 stage tumors [2]. Studies have shown that some patients in the high-risk group and those with stage III CRC may benefit from adjuvant chemotherapy [4]. To better individualize the treatment strategy, it is important to identify relevant and informative biomarkers.

Whether or not the tumor has a defect in the DNA repair mechanisms, such as mismatch repair genes, may be of prognostic value. Tumors can be sub-grouped according to microsatellite instability (MSI) status: i.e., as microsatellite—stable (MSS), microsatellite instability—low (MSI-L), or microsatellite instability—high (MSI-H). MSI-H is associated with a better prognosis in the early stages of CRC, as well as a worse response to 5-FU-based chemotherapy [5].

The enzyme thymidine phosphorylase is encoded by the gene *TYMP* [6]. *TYMP* has a proangiogenic character and has been shown to cause resistance to apoptosis [7, 8]. In CRC, *TYMP* is often elevated compared with non-neoplastic tissues, and its high level may correspond to a poor prognosis [6, 9]. Epithelial cells, as well as stromal cells, thrombocytes, endothelial cells, and tumor-infiltrating macrophages express *TYMP*. In *TYMP*-positive CRC, high macrophage infiltration correlates with worse prognosis [10].

Depending on stimuli, monocytes may develop into traditional M1-polarized macrophages, which are bactericidal, or into M2-polarized macrophages with proangiogenic and anti-inflammatory properties. In colorectal tumors, a higher M2/M1 macrophage ratio correlates with a worse prognosis [11, 12]. The cluster of differentiation 163 (CD163) protein is a macrophage-specific hemoglobin scavenger receptor used for identification of M2-polarized macrophages among tumor-associated macrophages (TAMs) [13, 14]. Increased levels of TAM have been observed in stages III–IV compared with stages I–II, and correspond to a more aggressive CRC [15]. Controversially, the strong infiltration of TAM in CRC has also been considered as a predictor of low tumor grade and less lymph node metastasis [16]. Furthermore, the location of TAM relative to the tumor epithelium and stroma is of interest because high levels of TAM in the tumor epithelium indicate a worse prognosis [17].

The tumor microenvironment plays a crucial role in the pathogenesis of CRC. A better understanding of the relationship between gene expression levels in different cell types in the microenvironment and tumorigenesis is needed [18, 19]. Stromal cells stimulate the tumor’s invasive and metastatic abilities. Therefore, the heterogenous composition of stromal cells and tumor epithelial cells may by itself be of prognostic value. Gene expression analysis is usually conducted with microdissected tumor tissues consisting of epithelial cells, thus excluding the influence of stromal cells. In macrodissected tumor tissues, both epithelial cells and stromal cells are included. However, it has been shown that the RNA yield from stromal cells is lower than that from epithelial cells, which means that the contribution of the stroma may have a minor effect on the gene expression profile [20]. Thus, the effect of *TYMP* and *CD163* gene expression on CRC carcinogenesis may vary depending on whether these genes are mostly expressed in tumor epithelial or stromal cells [10]. For example, in patients with primary, operable CRC, a high stromal *TYMP* gene expression has been shown to be associated with a favorable prognosis [7]. Whether macrodissected tissue is the best choice for analysis of gene expression, or if more accurate results will be obtained from microdissected tissues separated into epithelial and stromal cells, is presently unknown.

The aim of study was to identify the potential association between *TYMP* and *CD163* gene expression and relapse-free survival (RFS), along with the clinicopathological factors of patients with stage II CRC, as well as the variation in expression in macro- and microdissected tumor tissues, the latter being divided into tumor epithelial cells and stroma cells.

## Materials and methods

### Study population

From 2002 until 2015, 1105 patients underwent surgery for CRC stage II at the Sahlgrenska University Hospital/Östra, Gothenburg, Sweden. Stage II CRC was defined as tumor growth through the muscularis propria into the subserosa or through all layers of the colon possibly invading nearby organs [21], no presence of tumor cells in regional lymph nodes or near the colon, and no distant metastases. All radically resected stage II patients who relapsed within 5 years of follow-up, and did not receive neoadjuvant treatment, were identified and included in the study if tissue samples could be retrieved (*n* = 104). These patients were matched according to tumor stage, tumor differentiation, and age to 208 patients who were relapse free after a 5-year follow-up. Two patients from this control group were excluded due to unmeasurable gene expression.

### Macro- and microdissection of tumor tissues

Macroscopically dissected tumor tissues were snap frozen in liquid nitrogen and stored at –80 °C until further analysis. Formalin-fixed tumor tissues were microdissected and separated into tumor epithelial cells and stromal cells. If the tumor cell area to be analyzed was homogenous and large enough (≥ 80% tumor cells), a scalpel blade was used to manually collect the cells. Otherwise, tumor and/or stroma cells were microdissected using the PALM MicroBeam microscope (Carl Zeiss) at the CCI, Core Facilities, University of Gothenburg, Sweden. In 26 of the tumors, the stroma was lacking completely or was present in a very small area and, thus, could not be excised.

### Total RNA extraction, cDNA preparation, and real-time PCR

Total RNA was isolated from snap-frozen tumor biopsies (10–30 mg) using the RNeasy kit (Cat # 74104, Qiagen, Sollentuna, Sweden) according to the manufacturer´s instructions. Total RNA was also isolated from formalin-fixed paraffin-embedded (FFPE) 10 µm sections using the RNeasy FFPE kit (Cat # 73504, Qiagen, Sollentuna, Sweden) according to the manufacturer’s instructions. The TissueLyser (Qiagen, Hilden, Germany) was used to disrupt and homogenize the tissue. Conditions for cDNA synthesis are presented in Supplemetary file 1. Real-time qPCR was performed using the 7500 Fast Real-time PCR system (Applied Biosystems, Foster City, USA). Assay details and PCR conditions are described in Supplementary file 2.

### Microsatellite status

DNA was isolated from snap-frozen tumor tissues using an All-prep DNA/RNA mini kit (Cat # 80204 Qiagen, Sollentuna, Sweden), or from FFPE tumor tissue using an All-prep DNA/RNA FFPE kit (Cat # 80234; Qiagen, Sollentuna, Sweden). The MSI status was analyzed using the MSI Analysis System, version 1.2 (Promega, Madison, USA), which examined five microsatellites. The PCR was run on the Perkin-Elmer Gene Amp PCR system 9600 Thermal Cycler (Perkin Elmer, USA) according to the manufacturer’s instructions using 2 ng of DNA. The MSI markers were detected on an ABI prism 3730 instrument at KI Gene using the PowerPlex 4C matrix Standard (Cat # DG4800; Applied Biosystems, USA). MSI was defined as peak alterations in the marker electropherogram when tumor tissue was compared with matching mucosa. When more than one marker showed instability, the tumor was defined as MSI-H. If only one marker showed instability, it was defined as MSI-L. If no instability was detected, the tumor was designated as MSS.

### Filter-dense multicolor microscopy

Filter-dense multicolor microscopy (FDMM) was used to visualize the distribution of TYMP and CD163. FDMM is an enhanced multifluorescence setup, which enables the visualization of several proteins simultaneously in one tissue sample [22]. The FFPE colorectal tumor tissues were cut into 4-µm-thick sections, deparaffinized with xylene, and rehydrated with an ethanol series (100%, 70%, 50%). Antigen retrieval was carried out in a pH 9 buffer (Agilent DAKO, Santa Clara, USA) in a pressure cooker. Tissue sections were stained with an anti-EpCAM antibody (VU1D9 LSBio, Seattle USA) for 1 h at RT. Tyramide amplification with Opal570 was applied according to the manufacturer’s instructions (Perkin Elmer, Waltham, USA). Antibodies were stripped away by incubating the slides in pH 9 buffer at a high temperature (just before boiling) in the microwave for 5 min. After cooling to room temperature, the tissue sections were stained with an anti-CD163 antibody (10D6, Novus Bio, Centennial, USA) for 1 h at RT, followed by tyramide amplification with Opal520. Subsequently, the tissue sections were stained with an anti-thymidine phosphorylase antibody (abcam180783, Cambridge, UK) at RT for 1 h, followed by anti-rabbit-AF647 Fab_2_ (Jackson Immuno West Grove, USA) at RT for 40 min. The tissues were mounted on the DAPI ProLong Diamond Antifade and were scanned with the Metasystems Scanner (Axio Imager.Z2 Microscope (Zeiss), 20 ×, Oberkochen, Germany) [22].

### Statistical analysis

All statistical analyses were conducted using the commercial software JMP Pro 13.1.0 (SAS Institute Inc. Cary, NC, USA). Descriptive statistics and *t* test/ANOVA were used to evaluate the data. Values were presented as means ± standard deviations (SD), or as medians and ranges. The ΔΔC_t_ method was applied to calculate the gene expression values, which were then transformed logarithmically, as the data were not normally distributed. Contingency tables with the nonparametric Chi-square/Kruskal–Wallis test was used to assess the differences between the groups. The Pearson’s correlation coefficient (*r*) was used to compare the sets of continuous parameters measured in the same tissue. RFS was defined as the time period from primary surgery to any recurrence of CRC, thus censoring death of any cause. To assess the putative relation of classical risk factors and gene expression on outcome, in terms of hazard ratios with 95% confidence intervals, univariate Cox proportional hazards regression analysis was applied. A multivariate proportional hazards regression analysis was used to adjust for possible confounding factors. The Wald test was used to evaluate significance in multivariate analyses. *P* value < 0.05 was considered significant. No correction for multiple testing was done.

## Results

### Patient and tumor characteristics

Patient and tumor characteristics are presented in Table 1. As shown, there was an even gender and age distribution between the relapse and relapse-free groups. Two hundred and sixty-seven patients had colon cancer; 41 had rectal cancer; and 2 had tumors in both the rectum and the colon. Of all the patients with colon cancer, 140 had tumors on the right side, whereas 127 had tumors on the left side. There was no significant difference in tumor location between the two groups. Twenty patients received adjuvant chemotherapy: 8 from the relapse group and 12 from the relapse-free group. Patients receiving adjuvant chemotherapy had as expected a higher degree of high-risk features: 75% had a T4 tumor; 30% had low tumor differentiation; and 20% had emergency surgery. Adjuvant chemotherapy was given with 5-FU/leucovorin (FLV) as single treatment (*n* = 12) or as combination treatment (*n* = 8).

**Table 1:** Characteristics of the study population

	Relapse (*n* = 104)	No relapse (*n* = 206)	All patients (*n* = 310)
Age, median (IQR)	70 (60–79)	70 (61–78)	70 (61–78)
Gender, *n* (%)			
Female	50 (48.1)	103 (50.0)	153 (49.4)
Male	54 (51.9)	103 (50.0)	157 (50.6)
Differentiation, *n* (%)			
Well/moderate (G1–G2)	89 (85.6)	171 (83.0)	260 (83.9)
Poor (G3–G4)	11 (10.6)	22 (10.7)	33 (10.6)
Mucinous	4 (3.8)	13 (6.3)	17 (5.5)
Tumor location, *n* (%)			
Colon	84 (80.8)	183 (88.8)	267 (86.1)
Rectum	20 (19.2)	21 (10.2)	41 (13.2)
Colon and rectum	0	2 (1.0)	2 (0.64)
No. of examined lymph nodes, median (IQR)	19 (14–23)	23 (18–27)	21 (16–26)
T-stage, *n* (%)			
T3	81 (77.9)	190 (92.2)	271 (87.4)
T4	23 (22.1)	16 (7.8)	39 (12.6)
MSI status (%)^a^			
MSI-H	15 (15)	45 (22)	60 (19.8)
MSS/MSI-L	84 (85)	159 (78)	243 (80.2)

*IQR* interquartile range, *MSI* microsatellite instability, *MSI-H* microsatellite instability—high, *MSS* microsatellite—stable, *MSI-L* microsatellite instability—low, *n* number of patients

^a^MSI status could not be obtained for seven patients

### Microsatellite status

The results of the MSI analysis are presented in Table 1. Sixty patients (19.8%) had MSI-H; 4 (1.3%) MSI-L; and 239 (78.9%) MSS tumors. Out of the 267 colon cancer patients, 23% had MSI-H tumors. All MSI-H tumors were located in the colon, of which 50 were on the right side and 10 on the left. There was no association between MSI and age or T-stage, but more female than male patients had MSI-H tumors (*p* < 0.01). There was a difference in MSI status according to tumor differentiation: 53% of the mucinous tumors, 50% of the well/moderately differentiated, but only 14% of the poorly differentiated tumors were MSI-H (*p* < 0.01).

### *TYMP* gene expression

The mean *TYMP* gene expression in macrodissected tumor tissues (mac*TYMP*), microdissected tumor epithelial cells (tec*TYMP*), and microdissected tumor stroma (stroma*TYMP*) was 0.52 ± 0.56, 0.24 ± 0.23, and 0.32 ± 0.36, respectively. There was a positive correlation between mac*TYMP* and tec*TYMP* (*r* = 0.35; *p* < 0.01) and between stroma*TYMP* and tec*TYMP* (*r* = 0.51; *p* < 0.01); however, there was no correlation between mac*TYMP* and stroma*TYMP* (*r* = 0.13; NS). *TYMP* gene expression did not correlate with age, gender, or T-stage, nor with relapse variables (Table 2).

**Table 2:** TYMP expression according to pathological characteristics, MSI status, and relapse in stage II colorectal cancer

	*n*	macTYMP^d^	*p* value	*n*	tecTYMP^d^	*p* value	*n*	stromaTYMP^d^	*p* value
T-stage									
T3	235	0.50 ± 0.53		268	0.24 ± 0.23		247	0.32 ± 0.37	
T4	27	0.69 ± 0.77	NS	39	0.26 ± 0.21	NS	35	0.29 ± 0.27	NS
Tumor localization^a^									
Colon	221	0.55 ± 0.59		264	0.25 ± 0.24		242	0.34 ± 0.38	
Rectum	39	0.33 ± 0.26	< 0.01	41	0.17 ± 0.11	NS	38	0.20 ± 0.17	< 0.01
Tumor localization in colon									
Right-sided colon	108	0.62 ± 0.68		140	0.28 ± 0.25		131	0.35 ± 0.42	
Left-sided colon	113	0.48 ± 0.48	< 0.05	124	0.22 ± 0.23	< 0.01	111	0.33 ± 0.33	NS
Differentiation^b^									
Well/moderate (G1–G2)	223	0.44 ± 0.35		257	0.21 ± 0.20		236	0.29 ± 0.34	
Poor (G3–G4)	26	1.07 ± 1.20	< 0.01	33	0.48 ± 0.32	< 0.01	32	0.48 ± 0.39	< 0.01
Mucinous^b^	13	0.80 ± 0.85	< 0.01	17	0.28 ± 0.17	< 0.01	14	0.48 ± 0.55	< 0.01
MSI status^c^									
MSI-H	49	0.71 ± 0.63		60	0.30 ± 0.26		58	0.39 ± 0.50	
MSS/MSI-L	212	0.47 ± 0.53	< 0.01	240	0.23 ± 0.22	< 0.05	217	0.30 ± 0.32	NS
Relapse									
Yes	75	0.48 ± 0.46		103	0.25 ± 0.23		93	0.29 ± 0.29	
No	187	0.54 ± 0.59	NS	204	0.24 ± 0.23	NS	189	0.33 ± 0.39	NS

*SD* standard deviation, *MSI* microsatellite instability, *MSI-H* microsatellite instability—high, *MSS* microsatellite—stable, MSI-L: microsatellite instability—low, *NS* not significant, *TYMP* thymidine phosphorylase, *macTYMP* *TYMP* expression in tumor sample, *tecTYMP* *TYMP* expression in tumor epithelial cells, *stromaTYMP* *TYMP* expression in stroma

^a^Two tumors with localization in both the colon and rectum were excluded

^b^Mucinous tumors were compared with highly/moderately and poorly differentiated tumors

^c^Upon exclusion of the 20 patients that received adjuvant chemotherapy, there was no significant difference in terms of MSI status

^d^Values are expressed as the mean ± SD

The gene expression of *TYMP* was higher in colon tumors compared to rectal tumors, and both mac*TYMP* and tec*TYMP*, but not stroma*TYMP* expression was significantly higher in right-sided compared to left-sided colon tumors (Table 2). Poorly differentiated tumors had a higher mean *TYMP* gene expression compared with well/moderately differentiated tumors, regardless of sampling method. The *TYMP* gene expression in mucinous tumors was higher than well/moderately but lower than poorly differentiated tumors, with the exception of stroma*TYMP* (Table 2). The mean expression of both mac*TYMP* and tec*TYMP* was higher in MSI-H compared to MSI-L/MSS tumors; however, there was no difference in stroma*TYMP* expression according to MSI (Table 2).

### *CD163* gene expression

The mean *CD163* gene expression in macrodissected tumor tissues (mac*CD163*), microdissected tumor epithelial cells (tec*CD163*), and microdissected tumor stroma (stroma*CD163*) was 0.13 ± 0.25, 0.06 ± 0.09, and 0.11 ± 0.22, respectively. There was no correlation between mac*CD163* and tec*CD163* (*r* = 0.12; *NS*), but there was a weak correlation between stroma*CD163* and tec*CD163* (*r* = 0.14; *p* < 0.05), and between mac*CD163* and stroma*CD163* (*r* = 0.22; *p* < 0.01). *CD163* gene expression did not correlate with age, gender, tumor location, or relapse variables (Table 3). There was no difference in stroma*CD163* or mac*CD163* expression with regard to the T-stage.

**Table 3:** CD163 expression according to pathological characteristics, MSI status, and relapse in stage II colorectal cancer

	*n*	macCD163^d^	*p* value	*n*	tecCD163^d^	*p* value	*n*	stromaCD163^d^	*p* value
T-stage									
T3	233	0.11 ± 0.21		265	0.056 ± 0.072		244	0.12 ± 0.23	
T4	27	0.25 ± 0.50	NS	39	0.11 ± 0.15	< 0.05	35	0.075 ± 0.099	NS
Tumor localization^a^									
Colon	220	0.14 ± 0.27		262	0.062 ± 0.087		239	0.11 ± 0.23	
Rectum	38	0.067 ± 0.082	NS	40	0.061 ± 0.086	NS	38	0.097 ± 0.15	NS
Tumor localization in colon									
Right-sided colon	108	0.15 ± 0.30		138	0.062 ± 0.081		128	0.11 ± 0.18	
Left-sided colon	112	0.13 ± 0.25	NS	124	0.063 ± 0.094	NS	111	0.11 ± 0.28	NS
Differentiation^b^									
Well/moderate (G1–G2)	221	0.11 ± 0.23		255	0.058 ± 0.088		234	0.10 ± 0.21	
Poor (G3–G4)	26	0.21 ± 0.37	NS	32	0.10 ± 0.082	< 0.01	31	0.16 ± 0.28	< 0.05
Mucinous^b^	13	0.27 ± 0.32	NS	17	0.050 ± 0.042	< 0.01	14	0.14 ± 0.24	NS
MSI status									
MSI-H	49	0.20 ± 0.37		59	0.058 ± 0.056		57	0.13 ± 0.20	
MSS/MSI-L	210	0.11 ± 0.22	0.05	238	0.063 ± 0.093	NS	215	0.10 ± 0.23	< 0.05
Relapse^c^									
Yes	75	0.13 ± 0.26		100	0.071 ± 0.092		89	0.085 ± 0.13	
No	185	0.13 ± 0.25	NS	204	0.057 ± 0.084	NS	190	0.12 ± 0.25	NS

*SD* standard deviation, *MSI* microsatellite instability, *MSI-H* microsatellite instability—high, *MSS* microsatellite—stable, *MSI-L* microsatellite instability—low, *NS* not significant, *CD163* cluster of differentiation 163, *macCD163* *CD163* expression in tumor sample, *tecCD163* *CD163* expression in tumor epithelial cells, *stromaCD163* *CD163* expression in stroma

^a^Two tumors with localization in both the colon and rectum were excluded

^b^Mucinous tumors were compared with highly/moderately and poorly differentiated tumors

^c^Upon exclusion of the 20 patients that received adjuvant chemotherapy, tecCD163 expression was significantly higher in the relapse group (*p* < 0.05)

^d^Values are expressed as the mean ± SD

However, T4-tumors had a higher tec*CD163* gene expression compared with T3-tumors (Table 3)*.* There was also a significant difference between tec*CD163* and stroma*CD163* in terms of tumor differentiation. The mucinous tumors expressed lower tec*CD163* compared to well/moderately and poorly differentiated tumors. Mac*CD163* and stroma*CD163* gene expression was higher in MSI-H compared to MSI-L/MSS tumors; however, there was no difference in tec*CD163* according toMSI status (Table 3).

**Fig. 1:**
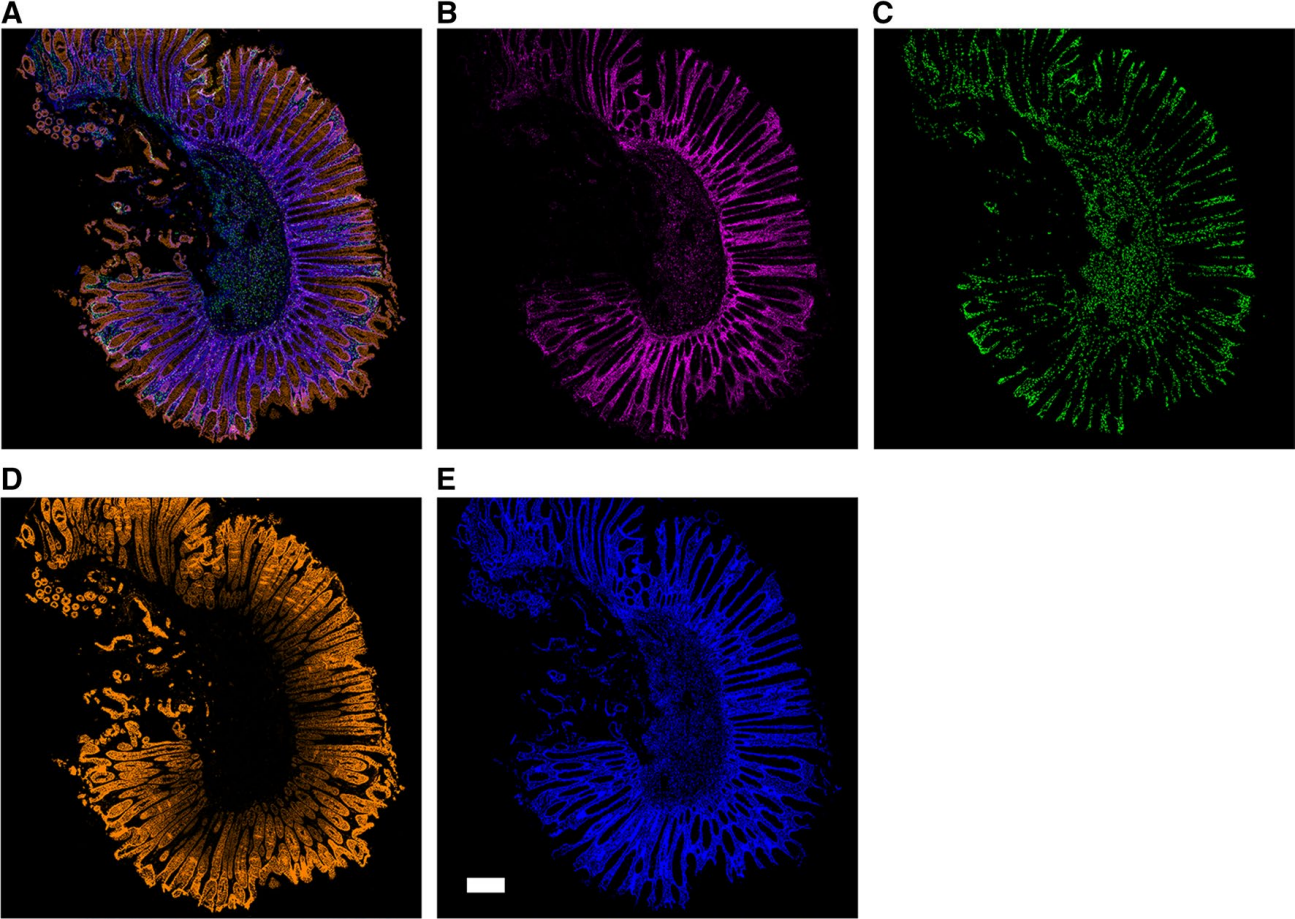
Filter-dense multicolor microscopy of stage II colorectal cancer. 500 μm scale bar. **a** Merged image, **b** thymidine phosphorylase (magenta), **c** CD163 macrophages (green), **d** tumor epithelium immunolabeled with antibodies against EpCAM (orange), **e** nuclei counterstained with DAPI (blue)

There was a positive correlation between TYMP and CD163 (*p* < 0.01) expression, comparing macTYMP with macCD163 (r = 0.37), tecTYMP with tecCD163 (r = 0.26) and stromaTYMP with stromaCD163 (r = 0.45). To visualize the distribution of TYMP and CD163-expressing macrophages in the tumor, the enhanced multifluorescence setup FDMM was performed (*n* = 12). FDMM revealed that TYMP and CD163 protein expression was heterogenous within and between samples. FDMM also showed that CD163 protein expression was not detected within, but near the tumor cells (Fig. 1).

### *TYMP* and *CD163* gene expression in relation to other risk factors

Cox regression univariate analysis of known risk factors showed that the tumor location, number of examined lymph nodes, T-stage, and whether the surgery was planned or acute were risk factors associated with RFS (Table 4). However, in the multivariate analysis, only T-stage and planned/acute surgery were found to be independent of other covariates included in the model. More advanced T-stage and acute surgery were associated with worse RFS. Tec*TYMP* expression was als an independent variable associated with RFS in the multivariate analysis. The risk of relapse increased with increased expression of *TYMP* in the epithelium.

**Table 4:** Association of covariates with relapse-free survival in stage II colorectal cancer

	Univariate			Multivariate		
	HR	95% CI	*p*	HR	95% CI	*p*
Age	1.01	0.98–1.02	NS	1.01	0.99–1.04	NS
Gender						
Female	1			1		
Male	1.11	0.75–1.64	NS	1.43	0.81–2.60	NS
Differentiation						
Well/moderate	1			1	0.39–9.74	
Poor	0.77	0.38–1.41		0.84	0.28–2.22	
Mucinous	0.66	0.20–1.59	NS	0.68	0.10–2.58	NS
Tumor location*						
Colon	1			1		
Rectum	1.94	1.16–3.1	< 0.05	1.88	0.93–3.70	NS
No. of examined lymph nodes	0.94	0.92–0.97	< 0.05	1	0.96–1.03	NS
T-stage						
T3	1			1		
T4	2.27	1.39–3.58	< 0.05	2.60	1.05–5.84	< 0.05
Planned surgery						
Yes	1			1		
No	3.5	2.05–5.61	< 0.05	5.44	1.29–19.22	< 0.05
Adjuvant chemotherapy						
No	1			1		
Yes	1.10	0.46–2.20	NS	1.26	0.33–6.4	NS
MSI status						
MSI-H	1			1		
MSI-L/MSS	1.55	0.92–2.79	NS	1.55	0.33–6.40	NS
TYMP^a^						
Log macTYMP	0.82	0.62–1.08	NS	0.69	0.46–1.05	NS
Log tecTYMP	1.06	0.85–1.33	NS	1.66	1.09–2.56	< 0.05
Log stromaTYMP	0.86	0.69–1.07	NS	0.92	0.65–2.56	NS
CD163^a^						
Log macCD163	1.04	0.89–1.21	NS	1.03	0.83–1.28	NS
Log tecCD163	1.15	0.98–1.34	NS	1.10	0.84–1.45	NS
Log stromaCD163	0.93	0.79–1.09	NS	0.95	0.75–1.23	NS

Patients were matched by age, tumor stage, and differentiation, and the number of patients included in the univariate analysis were the same as in Tables 1, 2, 3, whereas 225 patients were included in the multivariate analysis

*CI* confidence interval, *CD163* cluster of differentiation 163, *Log macCD163* logarithmized *CD163* expression in tumor sample, *Log tecCD163* logarithmized *CD163* expression in tumor epithelial cells, *Log stromaCD163* logarithmized *CD163* expression in stroma, *HR* hazard ratio, *NS* not significant, *MSI* microsatellite instability, *MSI-H* microsatellite instability—high, *MSS* microsatellite—stable, *MSI-L* microsatellite instability—low, *TYMP* thymidine phosphorylase, *Log macTYMP* logarithmized *TYMP* expression in tumor sample, *Log tecTYMP* logarithmized *TYMP* expression in tumor epithelial cells, Log *stromaTYMP* logarithmized *TYMP* expression in stroma

*When excluding adjuvantly treated patients (*n* = 20), multivariate analysis showed that patients with rectal tumors had an increased risk of relapse (*p* < 0.05)

^a^Gene expression values were not normally distributed and therefore, logarithmized in the statistical analysis

## Discussion

In this study, we analyzed *TYMP* and *CD163* expression in micro- and macrodissected tumor tissue from 312 patients with stage II CRC. The effect of an active gene within a tumor may depend on whether it is expressed in stromal or epithelial cells and to what extent. Comparing micro- and macrodissected tumor tissues, the highest *TYMP* gene expression was found in macrodissected tissues, including both stromal and epithelial cells. There was a correlation between mac*TYMP* and tec*TYMP*, suggesting that mac*TYMP* could be used as a surrogate for gene expression in tumor epithelial cells. These results were in concordance with those of a previous study comparing mRNA levels in micro- and macrodissected tissues. The authors concluded that mRNA levels of stromal cells were low, and reliable tumor-specific gene expression profiles could be obtained from macrodissected tissues [20]. However, in this study, multivariate analysis showed that only tec*TYMP* was significantly associated with RFS. This association would not have been detected if only mac*TYMP* had been analyzed. The tec*TYMP* expression was independent of the other two covariates associated with RFS namely, acute surgery and T4-tumors, which are known risk factors for CRC [2]. However, tec*TYMP* was not significant in the univariate analysis, which suggests that there were interactions between *TYMP* gene expression in epithelial cells and other risk factors. There was also a positive correlation between tec*TYMP* and stroma*TYMP*, possibly reflecting the overall *TYMP* gene expression in poorly differentiated tumors.

Although some studies evaluating TYMP as a predictive marker for chemotherapy have shown comparable results between microdissected tumor epithelial cells and macrodissected tissues, other studies show contradicting results [7, 20]. For example, when the expression levels of several 5-FU-related genes in micro- and macrodissected tumor tissues of patients with locally advanced rectal cancer who subsequently received radiotherapy were compared, a significant difference in *TYMP* gene expression was found [19]. The authors concluded that microdissection of tumor tissues after irradiation was important because of the changed tumor/stroma ratio induced by irradiation. However, in the present study no patient received radiotherapy, and there was a difference between macTYMP and tecTYMP which might indicate the importance of microdissection also in non-radiated CRC.

TYMP is expressed not only in the tumor epithelial cells but also in macrophages [6]. TYMP positive tumors and macrophage infiltration have previously been associated with a worse prognosis in CRC [10]. Furthermore, macrophages may polarize into an M2 macrophage subtype, considered as TAM, thus evolving pro-tumoral properties. In the study, it was possible to identify TAM both in micro- and macrodissected tissue by including the macrophage-specific marker CD163, and further, to stratify the influence of TAM in the tumor microenvironment and its correlation to TYMP expression. The results showed a positive correlation between TYMP and CD163 gene expression, both in macro- and microdissected tumor tissue. However, as visualized by FDMM, high TYMP protein expression could not be explained by high infiltration of CD163-positive macrophages expressing TYMP.

Several studies have shown that high CD163 expression correlates with worse survival, and higher CD163 expression has been reported in stages III–IV compared to earlier stages [13, 23, 24]. It has been suggested that in advanced cancer, the tumor epithelial cells can fuse with macrophages thereby adopting some of their abilities (such as migration) thus making them more prone to metastasize [13, 25]. If increased expression of CD163 is a late event during CRC development, this may explain the lack of association between *CD163* expression and recurrence or RFS in the present study on stage II CRC.

It is known that some tumor characteristics vary depending on the tumor location. For example, right-sided colon cancer most often has been associated with a worse prognosis, higher rate of MSI, BRAF mutations, and CpG island methylation [26, 27]. In contrast, left-sided CRC has been associated with a higher grade of p53 and KRAS mutations [26]. In the present study, almost 20% of the patients had MSI-H tumors, and as expected, these were preferentially located in the right side of the colon and more common among female than male patients. However, in support of previous studies in terms of survival, the MSI status was not of prognostic value for RFS [14, 28].

A limitation of the study was that non-microdissected sections of FFPE tissue were not analyzed as a complement to the macrodissected snap-frozen tissue. Another limitation was that we did not include any pan-macrophage marker for comparison of infiltration of M1 and M2 macrophages, which might be of importance, since a high M2/M1 ratio is associated with worse survival [24, 29]. It might be of interest to address these issues in future studies.

In conclusion, our findings revealed that CD163-expressing macrophages near tumor epithelial cells had high expression in poorly differentiated and T4 tumors. High *TYMP* gene expression was seen in poorly differentiated tumors, right-sided CRC, and MSI-H tumors. In tumor epithelial cells, high *TYMP* gene expression was associated with shorter RFS, independent of known risk factors. This emphasizes the need of further studies using microdissection to establish whether *TYMP* and *CD163* could add clinically relevant information to identify high risk stage II patients that could benefit from adjuvant chemotherapy.

## Supplementary Information

Below is the link to the electronic supplementary material.

Supplementary file1 (DOCX 14 KB)

Supplementary file2 (DOCX 15 KB)
