# Supplementary material for: Impact of thymidine phosphorylase and CD163 expression on prognosis in stage II colorectal cancer
Source: Clin Transl Oncol. 2022 May 14;24(9):1818–27. doi: 10.1007/s12094-022-02839-2 (PMC9338131; doi:10.1007/s12094-022-02839-2)
Supplement: Supplementary file 1 — Supplementary file1 (DOCX 14 KB) [file 12094_2022_2839_MOESM1_ESM.docx]

**Supplementary file 1**

**Clinical and Translational Oncology**

**Impact of Thymidine Phosphorylase and CD163 Expression on Prognosis in Stage II Colorectal Cancer**

Donia Kaidi, **^c^** Louis Szeponik, **^2^** Ulf Yrlid, **^2^** Yvonne Wettergren, **^1^** and Elinor Bexe Lindskog**^1^**

^1^ Surgical Oncology Laboratory, Department of Surgery, Institute of Clinical Sciences, Sahlgrenska University Hospital/Östra, the Sahlgrenska Academy at University of Gothenburg, SU Sahlgrenska 41345 Gothenburg, Sweden,

**^2^** Department of Microbiology and Immunology, University of Gothenburg, Medicinaregatan 7 41390 Gothenburg, Sweden

Donia Kaidi <https://orcid.org/0000-0002-6396-462X>

Louis Szeponik <https://orcid.org/0000-0002-8627-6969>

Ulf Yrlid <https://orcid.org/0000-0002-3431-6770>

Yvonne Wettergren <https://orcid.org/0000-0001-8660-7169>

*Corresponding author:*
Elinor Bexe Lindskog
Department of Surgery
Sahlgrenska University Hospital, Östra, SE-416 85 Sweden
Phone: +46 31 34 35 548; Fax: +46-31-3435930
[elinor.bexe-lindskog@surgery.gu.se](mailto:elinor.bexe-lindskog@surgery.gu.se)

<https://orcid.org/0000-0003-1466-1486>

**Conditions for cDNA synthesis**

cDNA was synthesized using the High Capacity cDNA Reverse Transcription kit (Thermo Fisher Scientific, Waltham, USA) and was run on a Maxygene II Thermal Cycler Therm-1000 (Axygene Scientific, Corning, USA) with the following program: 25°C for 10 min, 37°C for 120 min, 85°C for 5 min, and then kept at 4°C until PCR-ready. The presence of DNA was checked by using samples without the addition of reverse transcriptase.
