# Supplementary material for: Impact of thymidine phosphorylase and CD163 expression on prognosis in stage II colorectal cancer
Source: Clin Transl Oncol. 2022 May 14;24(9):1818–27. doi: 10.1007/s12094-022-02839-2 (PMC9338131; doi:10.1007/s12094-022-02839-2)
Supplement: Supplementary file 2 — Supplementary file2 (DOCX 15 KB) [file 12094_2022_2839_MOESM2_ESM.docx]

**Supplementary file 2**

**Clinical and Translational Oncology**

**Impact of Thymidine Phosphorylase and CD163 Expression on Prognosis in Stage II Colorectal Cancer**

Donia Kaidi, **^c^** Louis Szeponik, **^2^** Ulf Yrlid, **^2^** Yvonne Wettergren, **^1^** and Elinor Bexe Lindskog**^1^**

^1^ Surgical Oncology Laboratory, Department of Surgery, Institute of Clinical Sciences, Sahlgrenska University Hospital/Östra, the Sahlgrenska Academy at University of Gothenburg, SU Sahlgrenska 41345 Gothenburg, Sweden,

**^2^** Department of Microbiology and Immunology, University of Gothenburg, Medicinaregatan 7 41390 Gothenburg, Sweden

Donia Kaidi <https://orcid.org/0000-0002-6396-462X>

Louis Szeponik <https://orcid.org/0000-0002-8627-6969>

Ulf Yrlid <https://orcid.org/0000-0002-3431-6770>

Yvonne Wettergren <https://orcid.org/0000-0001-8660-7169>

*Corresponding author:*
Elinor Bexe Lindskog
Department of Surgery
Sahlgrenska University Hospital, Östra, SE-416 85 Sweden
Phone: +46 31 34 35 548; Fax: +46-31-3435930
[elinor.bexe-lindskog@surgery.gu.se](mailto:elinor.bexe-lindskog@surgery.gu.se)

<https://orcid.org/0000-0003-1466-1486>

**Real-time PCR conditions**

TYMP and CD163 transcripts were quantified using assay-on-demand Hs00157317_m1 and Hs00174705_m1, respectively (Thermo Fisher Scientific, USA). The endogenous control gene β-actin was used to compensate for variation in amount of RNA and to check the efficiency of the reverse-transcription reaction.

For quantification of *TYMP* gene expression, a multiplex PCR was performed by mixing 2x TaqMan Universal PCR master mix, no AmpErase UNG (Thermo Fisher Scientific, USA), with 20x TYMP assay-on-demand (Thermo Fisher Scientific, USA). The β-actin primers and probe were added to a final concentration of 100 nM, and the reaction mixture was then added to the cDNA.

For quantification of *CD163* gene expression, a singleplex PCR was performed by mixing 2x TaqMan Universal PCR master mix, no AmpErase UNG (Thermo Fisher Scientific, USA), with 20x CD163 assay-on-demand (Thermo Fisher Scientific, USA). The reaction mixture was then added to the cDNA. CD163 and β-actin were run in separate wells. The β-actin primers and probe were diluted with 2x TaqMan Universal PCR master mix, no AmpErase UNG, to a final concentration of 100 nM.

The following sequences for β-actin were used: probe, 5’-CCT GAA CCC CAA GGC CAA CCG-3’; forward primer, 5’-CGT GCT GCT GAC CGA GG-3’; and reverse primer, 5’-GAA GGT CTC AAA CAT GAT CTG GGT-3’.

Ten μl of each cDNA mixture were added in triplicates to the 96-well plate and analyzed. A positive control, negative control, and a dilution curve of a given sample were included in each run. The program started with incubation for 20 s at 95°C and thermal cycling of 40 cycles at 95°C for 3 s and at 60°C for 30 s. Quantitative data were calculated as the ΔΔCt values according to the manufacturers’ instructions (Thermo Fisher Scientific, USA).
